# Exploring salivary diagnostics in COVID-19: a scoping review and research suggestions

**DOI:** 10.1038/s41405-021-00064-7

**Published:** 2021-01-26

**Authors:** Priyanka Kapoor, Aman Chowdhry, Om Prakash Kharbanda, Deepika Bablani Popli, Kamini Gautam, Vikram Saini

**Affiliations:** 1 Orthodontics, Faculty of Dentistry, Jamia Millia Islamia, New Delhi, India; 2 Oral Pathology & Microbiology, Faculty of Dentistry, Jamia Millia Islamia, New Delhi, India; 3 Dr. C.G. Pandit National Chair of ICMR, Department of Plastic Surgery, All India Institute of Medical Sciences, New Delhi, India; 4 Laboratory of Infection Biology and Translational Research, Department of Biotechnology, All India Institute of Medical Sciences, New Delhi, India

**Keywords:** Occupational health, Infection control in dentistry, Continuing professional development in dentistry

## Abstract

**Introduction:**

Molecular diagnostics for SARS-CoV-2 infection characteristically involves the sampling of the throat or nasopharyngeal swab (NPS). However, these procedures are invasive, require necessary skills for sample collection, cause patient discomfort, and are non-conducive for extensive scale testing. Saliva is increasingly being suggested as an alternate diagnostic sample in SARS‐CoV‐2 infection.

**Objectives:**

This scoping review was done with the objective of exploring the evidence on the role of saliva as an alternate diagnostic sample in SARS‐CoV‐2 condition.

**Methods:**

Thorough search of the literature in major databases was undertaken in June 2020 using free text and MESH terms, followed by PRISMA to identify 17 studies for data extraction.

**Results and conclusions:**

Evidence was summarised for study characteristics, salivary sampling characteristics, viral load, and longevity of virus in saliva. The literature supports that saliva offers a simple sample collection method compared to technique-sensitive NPS and has the advantage of point-of-care testing for initial screening in community or hospital-based set-up. The additional highlights of this review are heterogeneity in the current literature and the gaps in methodology. Therefore, a robust study design to generate higher levels of evidence has been proposed.

## Introduction

Coronaviruses (CoVs) belong to a group of zoonotic RNA beta-CoV that primarily circulate among animals but can infect humans too.^1,2^ CoV as described on cryogenic electron microscopic images bear crown-like spikes on its surface^3^ and have been classified into four groups, namely, alpha, beta, gamma, and delta CoV. The alpha and beta variety of CoV infects mainly the human’s and mammal’s respiratory, gastrointestinal, and central nervous system. Gamma and delta types of CoV infects mostly birds.^4,5^

Till December 2019, six CoVs were known to infect humans out of which two were a variety of alpha CoV [229E, NL63] and four were beta CoV [OC43, HKU1, Middle East respiratory syndrome (MERS), severe acute respiratory syndrome (SARS)].^2^ Additional strain of CoV (seventh) affecting humans emerged in Wuhan (Hubei province), China from the Huanan seafood wholesale market on December 8, 2019.^3,6^ This strain was named as “severe acute respiratory syndrome coronavirus 2” (SARS-CoV-2) or coronavirus disease 2019 (COVID-19) by International Committee on Taxonomy of Viruses and World Health Organisation (WHO), who proclaimed it as a pandemic on March 11, 2020.^7^

The molecular structure of virion of SARS-CoV-2 has a diameter ~50–200 nm^3^ comprises of four structural proteins: (1) Spike (S); (2) Envelope (E); (3) Membrane (M); and (4) the nucleocapsid (N). Of these, S protein has a high affinity for human angiotensin-converting enzyme 2 (hACE2) receptors in the host cells and aids in its subsequent entry into the human body.^8^ The interactions between the host ACE2 receptors and SARS-CoV spike protein have been implicated for human to human transmission of the virus,^9^ but a comprehensive mechanism of its binding to SARS-CoV-2, leading to pathological damage, requires further investigation.

Viral diagnosis has progressed tremendously with a multitude of recent and more accurate techniques ranging from laboratory testing to advanced point of care. Of the various modalities for the SARS-CoV-2 diagnosis, the most reliable test is reverse transcription polymerase chain reaction (RT-PCR) on nasopharyngeal swabs (NPSs).^10,11^ However, NPS necessitates the availability of skilled technical staff, causes significant patient discomfort during test sample collection, and is associated with a high risk of infection to healthcare workers (HCWs) involved.^12,13^ Additional factors like psychological fear of collection and inadequate sampling technique may diminish specimen quality and lower test sensitivity.

**Fig. 1:**
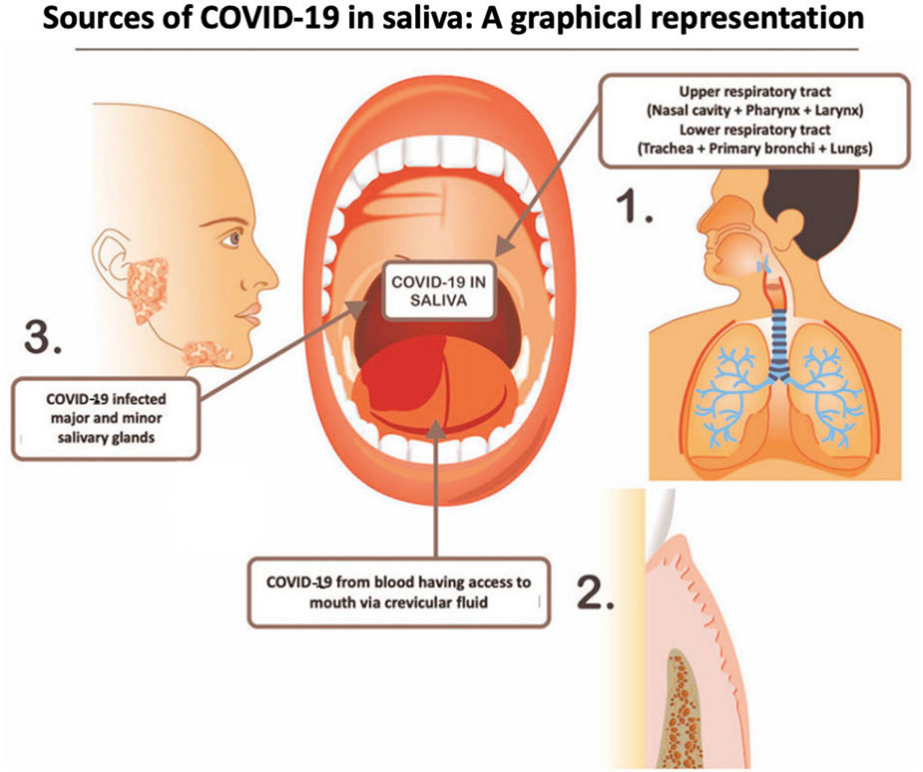
Sources of COVID-19 in saliva. A graphical representation showing three significant pathways for COVID-19 to reach saliva. (1) from the secretions of the upper and lower respiratory tract, (2) from the blood via gingival crevicular fluid and, (3) from the secretions of the infected major and minor salivary glands.

Saliva is suggested to cause human-to-human transmission via droplet infection and may be used as an alternative to NPS for SARS-CoV-2 detection. Sabino-Silva et al. have compiled and proposed three different pathways for COVID-19 for reaching the oral cavity (Fig. 1): first, from upper and lower respiratory tract as a source; second, from blood having access to the mouth via crevicular fluid; third, from infected major and minor salivary glands.^14^

Saliva may serve as a reliable alternative to NPS by offering advantages of self-collection of sample and countering issues like scarcity of NPS swabs and protective gear for concerned medical staff.^14,15^ Interestingly, some studies also suggest a positive result for SARS-CoV-2 in salivary specimens while NPS remains negative in paired samples.^16,17^

Hence, the current scoping review was planned to analyse the feasibility of saliva as a diagnostic sample for detecting SARS-CoV-2 infection. The objectives were to critically evaluate the current evidence related to salivary diagnostics in the progression of SARS-CoV-2 from an early stage of infection to recovery. The review also considers the strengths and shortcomings of salivary studies to deduce a robust study design to generate a higher level of evidence.

### Search strategy and selection criteria

**Fig. 2:**
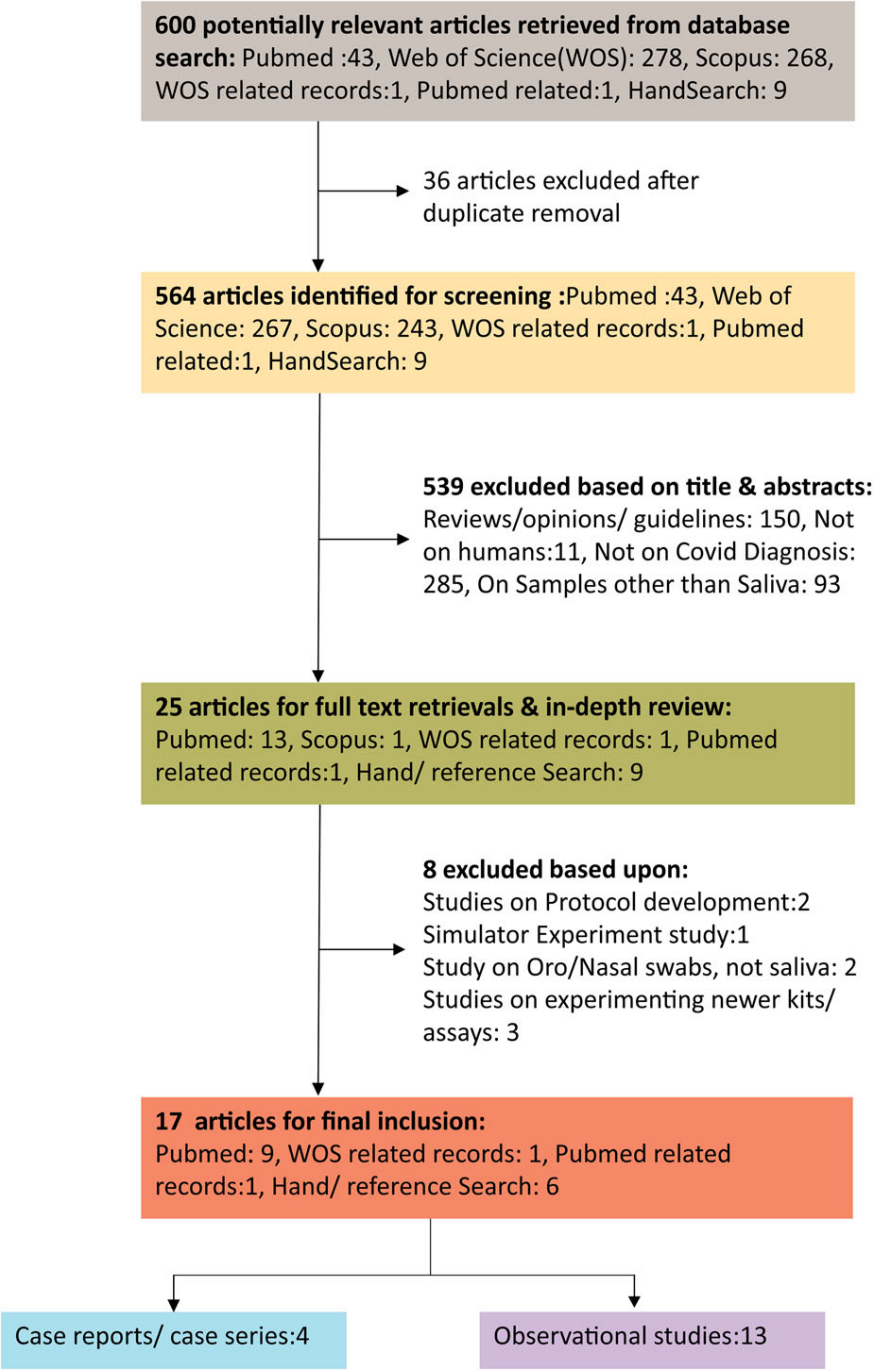
Search strategy. Preferred reporting Items for systematic reviews and meta-analysis (PRISMA) was employed to search the literature from different sources.

A scoping review of the literature was conducted to study the effective value of saliva samples for COVID-19 diagnosis. A thorough search of the literature was conducted in three databases Pubmed (P), Web of Science (WOS), and Scopus along with specialised COVID issues of PubMed (https://www.ncbi.nlm.nih.gov/sars-cov-2/), WHO (https://www.coronavirus.gov.https://www.coronavirus.gov), and International Association of Dental Research COVID Resource in June 2020, along with hand search (HS) and reference tracking. The terms used for the search were MESH terms “Saliva”, “Diagnosis”, and free text terms “COVID”, “SARS”, and “Corona”. The initial search revealed 43 articles in (P), 278 in WOS, 268 in Scopus along with 5 in (HS) and 2 in related search. The preferred reporting system of systematic reviews and meta-analysis (Fig. 2) criteria left us with 17 studies for data extraction. Using PICO (participants, intervention, comparator and outcome) criteria (Table 1), three researchers did data extraction individually, and the fourth researcher addressed any discordance.

**Table 1:** The participants, salivary, and results’ characteristic of the included studies.

S no.	Ref. no.	Author	Single/multicentric	Participant characteristics	Salivary sampling characteristics	Confirmatory test	Primary salivary outcome	Limitations
1	^16^	Wyllie et al. (2020)	Single	70 confirmed COVID-19 hospitalized patients (paired samples of NPS and saliva), mean age—61.4 yrs, 41 M/29 F, controls—495 asymptomatic HCWs, (paired samples—9), mean age—37.6 yrs	Early morning saliva, serial sampling at Av. every 2.9 d, quantity—1/3 of urine cup, self-collection	rRT-qPCR	Viral titres in saliva >NPS, serial specimens show a decrease in viral load from the day of symptom onset, atleast one HCW tested +ve for SARS-CoV-2 both in saliva and NPS, greater variability in quality of self colected NPS compared to saliva.	Only COVID-19 hospital inpatients with severe ds, no pre-symptomatic sample
2	^17^	Azzi et al. (2020)	Single	25, 2 gps: severe and very severe ds, mean age 61.5 +/− 11.2 yr, 17 M/8 F, no controls	Repeat sampling after 4 days (8 patients), drooling technique in saliva, collection by health personnel	rRT-PCR, Luna Universal qPCR Master Mix (New England BioLab)	Salivary detection in all 25 patients, Ct values range −18.12 to 32.23—less than a threshold of 33, no corr. with days from symptom onset, age, sex, and comorbidities, viral load in inverse corr. with inlflammatory index	No control, only severe disease patients
3	^18^	Tajima et al. (2020)	Single	1, COVID-19 hospitalized pateints, 71 yr M, no control	Saliva serial sample each day from 9 to 45 days post infection, both EMSS (from day 28 of ds onset) and DSS(till day 26 of ds onset), 600 µL quantity, using SMGNP, self-collection under supervision	RT-PCR	Detection of virus in saliva up to 37 d after symptom onset, even after symptoms subside. DSS sensitivity—25.0% and specificity—100%, EMSS sensitivity—66.7% and specificity—100%	One patient case report
4	^19^	Yoon et al. (2020)	Single	2, COVID-19 hospital inpatients, 46 and 65 yr, 2 F/0 M, no controls	Serial saliva sampling every 2 days from hospital days 1–9, additional samples after CLX mouthwash at 0 h(before), 1 h, 2 h, 4 h, self-collection	rRT- PCR, CFX96 (Bio-rad, Hercules, CA, USA) and PowerChek 2019-nCoV (Kogenebiotech, Seoul, Korea)	Viral load highest in NPS (7.49–8.41 log_10_ copies/mL), also elevated in saliva (6.63–7.10 log_10_ copies/mL), detection till day 6 of hospital admission (day 9 of illness-2nd patient ), a transient decrease in salivary viral load for 2 h using chlorhexidine MW	Sample size small, no controls (no gargling with saline)
5	^20^	Han et al. (2020)	Single	27-day-old neonate, mother (confirmed COVID-19), no controls	Serial sampling, health personnel	rRT-PCR kit (Kogene Biotech, Seoul, Korea)	Load of virus in NPS > saliva in early stages of the disease, fall below detectable levels in saliva in approximately 10 d	One neonate subject
6	^21^	Azzi et al. (2020)	Single	2, 71, and 64 yr M, severe disease, no controls	Patient 1: drooling technique, after 10 days of admission, health personnel. Patient 2: pipette collection, 26 and 30 days after an initial diagnosis of COVID-19, health personnel	RT-qPCR, Luna Universal qPCR Master Mix (New England Biolabs)	Detection in saliva till 10th d of hospital admission in patient 1 and till 26^th^ and 30^th^ d in patient 2 while NPS samples were −ve on same days	Only 2 patients, conditions of salivary collection not explained
7	^22^	Cheng et al. (2020)	Single	1 confirmed COVID-19, no control	Saliva sample, self-collection	rRT- PCR, LightMix Modular SARS and Wuhan CoV E-gene mix, LightCycler Multiplex RNA Virus Master Kit	Viral load in saliva >pooled NPS and throat swabs, environmental surveillance performed	One subject sample, no control
8	^23^	Zheng et al. (2020)	Single	96, COVID-19 inpatients, mild (22) and severe (74), median age—55 yr, 58 M/38 F, no controls	Deep cough without sputum, serial sampling, 1178 specimens (Av. sample range/patient (3–40), every day after admission, by health personnel	RT-qPCR, commercial kit (BoJie, Shanghai, China)	Decrease in detection rates from 95% (first wk of symptom onset) to 54% (fourth wk), median duration of virus and viral load in severe ds significantly >mild disease, viral load in respiratory samples >stool samples >serum	Sample quality questionable, testing on symptomatic patients only
9	^24^	Pasomsub et al. (2020)	Single	200, symptomatic patients attending acute respiratory infection clinic (181 NPS + throat swab −ve,19 NPS + throat swab +ve), median age 36 yr, 69 M/131 F, no controls	Saliva sample void of coughing, self-collection	RT-PCR, CFX96 (Bio- Rad, Hercules, CA, USA)	Sensitivity of diagnosis in saliva 84.2% and specificity 98.9%, PPV for saliva 88.9% and NPV 98.4%, κ coeff. (0.8)—strong agreement b/w saliva and NPS + throat swab	Symptomatic patient sample, no healthy controls
10	^25^	McCormick-Baw et al. (2020)	Single	156, suspected and confirmed COVID-19 from ED and inpatients, Av. age—47.8 yr, 90 M/66 F, no controls	1 mL saliva (not sputum) and NPS samples same day, self-collection under supervision	POCT, RT-PCR, Xpert Xpress SARS-CoV-2 assay (Cepheid, Sunnyvale, CA)	Viral load in NPS > saliva, overall positivity in paired NPS and saliva specimens—32.1%, +ve percent agreement 96% (47/49), −ve percent agreement 99% (1/106)	Single-centre cohort study
11	^26^	Wang et al. (2004)	Single	17 probable SARS-Cov-2, median age: 41 yr, 9 M/8 F, 12 healthy controls	Saliva and throat wash (by gargling 10 mL normal saline), Av. sampling day 4.8 (2–9 days range) of fever, in airborne isolation, by health personnel	rRT-qPCR	Paired samples—higher viral load in saliva than throat wash, linear corr. b/w viral load of saliva and throat wash, no corr. b/w sampling day and viral load	Sample size insufficient, only symptomatic patients
12	^27^	To et al. (2020)	Single	12, confirmed SARS-CoV-2, median age 62.5 yr, 7 M/5 F, 33 controls (NPS −ve)	Patient coughed up saliva in a sterile container. Sample collection was done 2 days (median) after hospitalization (range for sample collection 0–7 days). Serial salivary specimens in 6 patients, self-collection.	RT-qPCR, Quanti Nova SYBR Green kit (Qiagen)	Detection in initial saliva specimens—91.7%, high median viral load of first available saliva specimens, decline in levels in serial salivary specimens	Small sample, sample quality questionable
13	^28^	Williams et al. (2020)	Single	39 confirmed COVID-19, 50 controls (NPS RT-PCR −ve)	Pooling saliva in their mouth for 1–2 min and spit 1–2 mL, self-collection	Multiplex RT- PCR, Aus Diagnostics, Mascot, Australia	Detection rate in saliva—84.6%, viral load in NPS > saliva, inverse corr. of viral load with days of symptom onset	Age range and sex ratio not specified, clinical severity of disease not detailed.
14	^29^	Pisanic et al. (2021)	Single	33 confirmed COVID-19 with symptoms, 134 negative samples (pre-pandemic & participants of ongoing studies)	GCF (referred to as saliva) by gently brushing the gum line for 1–2 min, health personnel	Multiplex magnetic microparticle (bead)-based SARS-CoV-2 saliva IA	Salivary IgG—100% sensitivity at ≥10 d after onset of symptoms, salivary IgG (anti-RBD) IgG—100% specificity, temporal kinetics of IgM, IgG, IgA in saliva consistent with serum	Salivary data cross-sectional, temporal kinetics of saliva and impact of infection severity not evaluated, socio-demographic and medical history information not correlated
15	^30^	To et al. (2020)	Multicentric	23, (10 F/13 M) 2 gps: (1) Severe ds—Av. age—66 yr, 4 F/6 M, (2) Mild ds—Av. age 56 yr, 6 F/7 M, no controls	POP saliva, 173 specimens, serial sampling, early morning, self-collection/endotracheal intubation	RT-qPCR	High median viral load—at presentation, highest in first wk then decline, no corr. of intial viral load with days after symptom onset and comorbidities. Significant +ve correl. b/w age and peak viral load. Viral longevity in POP saliva—20 d or longer in 1/3 patients	Sample quality questionable, no healthy control
16	^31^	Chen et al. (2020)	Single	31, confirmed COVID-19 (15 M/16 F), median age 60.6 yr), no control	1.5 mL pure saliva collected from ductal openings, health personnel	RT- PCR, BioGerm Inc., Shanghai	Saliva detection rate—75% in critically ill patients, two significant oral-related symptoms of dry mouth and amblygeustia found in high proportion in COVID-19 patients	Small sample, no control
17	^32^	Chen et al. (2020)	Single	58 pairs—NPS + saliva, COVID-19 inpatients, median age 38 yr, 28 M/30 F, no controls	POP saliva, coughed up by clearing throat and spit 1 mL of saliva, same day sampling of saliva and NPS, early morning, self-collection	rRT-PCR,POCT Xpert Xpress SARS-CoV-2 assay (Cepheid, Sunnyvale, CA) E and N2 gene target	Detection rate in both NPS and saliva 84.5%, 10.3% in NPS only, and 5.2% in saliva only. Higher viral load in NPS than saliva, 100% concordance of POCT assay with rRT-PCR. N2 gene (>93%) showed greater detection than the E gene (<90%)	No healthy controls

The table depicts data extraction under majorly three parameters: Participant characteristics—Number, age and distribution of study and control participants, Salivary characteristics—Quality, mode, day, person for saliva collection and confirmatory test, Outcomes: Sensitivity, specificity of salivary sample, viral and antibody titres in saliva.

*+ve* positive, *−ve* negative, *anti-N* anti-nucleoprotein, *anti-RBD* anti-receptor-binding domain, *Av.* average, *b/w* between, *coeff* coefficient, *corr.* correlation, *Ct* cycle threshold, *d* day/s, *ds* disease, *DSS* daytime saliva sample, *ED* Emergency Department, *EMSS* early morning saliva sample, *F* female/s, *gps* groups, *HCW* healthcare worker, *h* hour/s, *IA* immunoassay, *M* male/s, *Ig* immunoglobulins, *min* minute, *ml* millilitre, *MW* mouthwash, *NPS* nasopharyngeal swab, *NPV* negative predictive value, *POCT* point-of-care test, *POP* postero-oropharyngeal, *PPV* positive predictive value, *RT-PCR* reverse transcriptase-polymerase chain reaction, *RT-qPCR* quantitative RT-PCR, *rRT- PCR* real-time RT-PCR, *SMGNP* Sugar chain-immmobilized magnetic gold nanoparticle, *yr* year/s, *wk* week/s.

## Results

This scoping review has broadly studied the presence of viral load in the saliva and its sensitivity in comparison to swab-based diagnostics. The results were used to establish the utility of a new sampling strategy, i.e. saliva-based molecular diagnostics in SARS-CoV-2.

### Study characteristics

The studies included in the review were mostly unicentric; a majority of them conducted in China. The sample size of 14 studies was <100, with 4 of them being case series/reports of 1 or 2 patients,^18–21^ 1 study evaluated saliva in a single subject,^22^ and 3 studies had sample nearing or above 100.^23–25^ RT-PCR was the most common technique utilised for identification of SARS-CoV-2. Twelve studies did not have control groups. Controls were used in 5 studies [(*n* = 12),^26^ (*n* = 33),^27^ (*n* = 50),^28^ (*n* = 9 paired samples),^16^ pre-pandemic salivary samples (*n* = 134).^29^

Both cross-sectional and longitudinal observational cohorts have been used in the current scoping review to assess SARS-CoV-2 viral load in salivary samples at different stages or days from disease onset. While few studies did serial salivary sample collection in the same patient at different observation points,^16–19,21,23,27,30^ one study evaluated salivary viral load in different patients at different days of symptom onset.^26^

### Viral load characteristics in serial sampling

Serial salivary specimens of 23 patients of severe and mild SARS-CoV-2 presented with a median of 5.2 log_10_ copies/mL viral load and showed the maximum load during the initial 1 week of hospitalisation, only to decline after 1 week.^30^ Another study, where serial samples were taken after a gap of 4 days, in 8 out of 25 severe and very severe disease patients showed Ct values (mean 27.16 ± 3.07) below a threshold value of 33, both in first salivary swab and the repeat sample.^17^ High log_10_ count was also observed in saliva in 2 patients (6.63 log_10_ copies/mL and 7.10 log_10_ copies/mL in patients 1 and 2, respectively) who underwent repeat sampling every 2 days from hospital day 1 to 9.^19^

Interestingly, in one study, samples were collected cross-sectionally from different patients at different days of disease rather than repeat sample of the same patients. The study showed similar results with the highest count of 6.38 × 10^8^ copies/mL in first week samples (average 4.8 days), before the development of lung lesions and slight fall by day 9.^26^

Another study identified the difference in viral load between severe and mild diseases with the virus levels being higher in severe than the mild disease (*p* = 0.03) and remained high until the third and fourth weeks.^23^ The mild disease pattern, however, followed the previous studies with high initial viral load in the first 1 week with a peak in salivary viral load in the second week, followed by a decrease. The severe disease pattern showed high levels even in the third and fourth weeks.^23^

The results of serial specimen studies, in general, revealed a trend of high salivary viral load during initial 1 week of onset of symptoms,^19,23,26,30^ but the viral load in endotracheal aspirate of few ambulatory patients did not decrease with time, indicating a continuous high viral load in the lower respiratory tract.^30^ Only one 65-year-old female patient with pneumonia on lopinavir/ritonavir 400 mg/100 mg treatment showed fall in viral load from day 1 of hospital admission (day 4 of illness), although the levels still remained consistently high till sixth day of hospital admission.^19^

Studies did not support change in initial viral load in saliva with period elapsed from symptom onset.^17,26,30^ Contrastingly, a single cross-sectional study analysing saliva and NPS samples from the COVID screening clinic reported an inverse relation of viral load with days from symptom onset.^28^

### Salivary/serum antibody response

The seropositivity was detected after 10 days of symptom onset with immunoglobulin G (IgG) values greater than IgM both for anti-nucleoprotein (NP) and anti-receptor-binding domain (RBD), which correlated with the virus neutralisation titre also used for retrospective diagnosis.^30^ One study also conducted serologiocal tests on specimens collected 28 days after symptom onsetand confirmed SARS-CoV-2 infection in 13 out of 17 patients.^26^ One study specifically studied the antibody responses in saliva (*n* = 33) and serum (*n* = 206) samples from SARS-CoV-2 patients. The authors reported 100% sensitivity of salivary anti-NP IgG at ≥10 days after onset of symptoms and 100% specificity of anti-RBD IgG, with their antibody response concurrent with serum.^29^

### Viral load sensitivity in saliva

Detection of SARS- CoV-2 virus in salivary/respiratory samples varied in studies with the duration from disease onset, quality of the sample (pure saliva or mixed with sputum/bronchopulmonary secretions), and disease severity. Studies showed high detection of the virus in saliva at 100% (25 severe disease patients,^17^ 96 patients of severe and mild disease),^23^ at 91.7% in 12 patients,^27^ and 75% in 4 critically ill patients.^31^ There was a gradual decrease in sensitivity from 95 to 54% from the first week to the fourth week of symptom onset, and the decrease is significant in severe compared to mild disease.^23^ Studies with paired saliva and NPS samples showed a high positive percent agreement of 84.5%^32^ and 96%^25^ in both the samples. The overall positivity of paired samples was found to be 32.1% in probable SARS-CoV-2 patients (50 out of 156).^25^

### Viral longevity in saliva

The longevity of the virus in salivary/respiratory sample of mild and severe disease patients have been investigated in 2 studies, which depict an average of 18–20 days.^23,30^ Of these, 1 study also distinguishes significantly prolonged detection of the virus in severe [21 days, interquartile range (IQR) 14–30 days] than the mild disease [14 days, IQR 10–21 days; *p* = 0.04].^23^ Another study on 2 patients documented the virus in saliva up to the 9th day of hospitalisation/13th day of illness.^19^ A case report in a 71-year-old patient reported virus after 37 days of symptom onset, despite the patient becoming asymptomatic.^18^ Similarly, 1 patient in a cohort of 12 hospitalised patients showed viral load shedding up to 11 days of hospitalisation.^27^ A singular study on neonate depicted undetectable viral load in saliva after 10–11 days as graphically depicted but remained detectable in NPS, stool, and urine.^20^

### Viral load quantification

Viral load was assessed by viral count as copies/mL, where 4 studies mentioned high initial log_10_ values (>5 log_10_),^19,26,27,30^ and 1 log_10_ copies/mL were labelled as undetectable.^30^ Other studies considered Ct value for viral load determination and categorised samples as negative when Ct values exceeded 33,^17^ 35,^31^ and 38.^16,23,24,31^ Few studies have used Ct values for calculating RNA copies (copies/mL), using a dilution of plasmid DNA for generating the standard curve.^16,19^ Specific gene targets have been compared in two studies on saliva. Chen et al. demonstrated slightly higher detection rate (93.1%) and Ct value, (IQR: 29.9–38.6) for N2 gene than E gene target (IQR 27.2–37.2), whereas McCormick-Baw et al. demonstrated average Ct values of 30.40 ± 9.67 for N2 gene and 26.10 ± 11.20 for E gene.^25,32^

### Saliva as an alternate to standard NPS sample

Studies have been done to compare SARS-Cov-2 detection in paired samples of saliva and NPS, the percentage of the total sample, viral load, or a combination of these parameters.^16,17,19–22,24,25,27,28,32^ Studies majorly revealed a higher viral load in NPS than in saliva specimens with lower Ct values and higher log_10_ count.^19,20,24,25,32^ The comparison of parameters in saliva and NPS have been outlined in Table 2. Two studies targeted point-of-care testing (POCT) in saliva.

**Table 2:** Comparison of parameters in saliva and nasopharyngeal swabs.

Parameters	Specific characteristics	Results
Gene targets	E and N2 gene targets	Earlier median Ct value in NPS than in saliva, statistically significant in one study—*p* = 0.0002,^32^ non-significant in other^25^
	ORF1ab and N genes	Lower median Ct value (32.0 and 30.5), in NPS than in saliva (32.7 and 31.8), for ORF1ab and N genes, respectively, though non-significant^24^
		Detection of ORF1ab and nCoV-N in NPS > saliva^31^
Mean viral titre	Salivary viral load > NPS	SARS-CoV-2 RNA copies in saliva (mean log_10_ 5.58) gretaer than NPS (mean log_10_ 4.93)^16^
		Viral load higher (5.9 × 10^6^ copies/mL) in saliva than pooled NPS and throat swabs (3.3 × 10^6^ copies/mL) in 1 patient out of 42 confirmed cases^22^
	Salivary viral load <NPS	Study on neonate: a steep difference in viral load level in NPS than in saliva in early stages of the disease^20^
		Significantly high viral titres with significantly low Ct values in NPS than in saliva at all time points^28^
Viral sensitivity	Positive results in saliva and not in NPS on same day collection	Multiple studies reported the finding in paired NPS and salivary samples: (*n* = 1),^25,28^ (*n* = 2),^17,24^ (*n* = 3)^32^
	Sensitivity in NPS > saliva	Saliva positive in 4 out of the 13 subjects tested positive by NPS^31^
		COVID-19 screening clinic: 39 out of 622 NPS samples tested PCR-positive (6.3%; 95% CI, 4.6–8.5%), and out of these 39 patients, 33 salivary samples tested SARS-CoV-2 positive (84.6%; 95% CI, 70.0–93.1%)^28^
		A single case report of two male patients: >60 years, showed positive salivary sample on day 10 and 26 after hospital admission, whereas 2 consecutive NPS samples came negative^21^
POCT	Viability of saliva for POCT	Two studies, one study showed high +ve percent agreement b/w NPS and saliva (96%, 47/49 positive samples)^25^ and high −ve percent agreement (99%, 1 sample +ve for saliva out of 106 samples −ve for NPS),^25^ the second study showed +ve testing of virus in both saliva and NPS (49/58), greater in NPS only [10.3% (6/58)] compared to saliva only [5.2% (3/58)]^32^

The parameters of gene targets, mean viral load, and sensitivity have been compared between saliva and NPS.

*+ve* positive, *−ve* negative, *b/w* between, *CI* confidence interval, *Ct* cycle threshold, *NPS* nasopharyngeal swab, *PCR* polymerase chain reaction, *POCT* point-of-care testing.

### Associations with salivary viral load

A significant positive correlation of age with peak viral load in saliva (*p* = 0.02)^30^ as well as the duration of the infection, which was found significantly increased in severe disease patients aged >60 years than <60 years (*p* = 0.01).^23^ A single study showed no correlation of Ct values with age (*p* = 0.34).^17^ Contrasting results related to sex predilection were obtained where one study showed no correlation of Ct values with sex (*p* = 0.31) or comorbidities,^17^ while another reported a significantly longer duration of the infection in men than in women in severe disease patients (*p* = 0.01).^23^

### Salivary sampling characteristics

Early morning saliva before tooth brushing and breakfast has been preferred as a test sample,^18,30,32^ as during night in the supine position, the nasopharyngeal and bronchopulmonary secretions get collected in the posterior oropharyngeal area. The secretions can be collected by deep cough,^23,30,32^ spitting,^16,18,28^ or gargling saline.^26^ Two of the studies used the drooling technique to collect saliva to eliminate the oropharyngeal secretions,^17,21^ while one study collected salivary swabs from the opening of the salivary gland duct.^31^ One study used a collective respiratory sample, including both saliva and sputum samples.^23^ Tajima et al. compared early morning saliva samples (EMSSs) with daytime saliva samples (DSSs) and found 66.7% sensitivity in EMSSs (4/6) compared to 25.0% (2/8) in DSSs, both EMSS and DSS had a similar specificity (100%), though the sensitivity of EMSS was much better than DSS.^18^ Avoidance of food, drink, tobacco, or gum for 30 min before saliva collection has been followed in one study.^25^ Room conditions of airborne isolation have also been considered in one study.^26^

Some clinical parameters of intervention have also been studied singularly, with 1 study testing the effect of chlorhexidine mouth wash (0.12%, 15 mL) for 30 s on viral load reporting a transient decrease in load in 2 h post gargling and then increase in 2–4 h.^19^

## Discussion

For molecular diagnostics of SARS-CoV-2, NPS or throat swabs are standard samples. However, collection of the throat swabs or NPS may induce sneezing, coughing, and expelling virus particles, which exacerbate health hazards for HCWs. Moreover, the collection of these swabs is a relatively invasive procedure that causes significant patient discomfort and may also induce bleeding in tonsils and posterior pharynx. NPS collection procedure is time-consuming, requires consumables, specialised set-up, and can be done by trained professionals only. Further, as the pandemic magnifies in intensity, the requirement of mass screening in densely populated locations, more so in poor and developing countries, is rising. Huge global demand for swabs is expected in the near future. With the above constraints of NPS, an alternate easy-to-use, less technique-sensitive, but reliable diagnostic sample is the need of the day.

Herein, based on a careful appraisal and interpretation of literature, we support the hypothesis that saliva can be a viable sample for the molecular diagnosis of SARS-CoV-2. Sufficient evidence has been generated in the present scoping review related to salivary specimens in SARS-Cov-2 for viral replication, longevity, sensitivity, specificity with other related viruses, and its practicality in the collection of specimens.

Tracking viral load can be vital in monitoring SARS-CoV-2 infections, risk assessment for infectivity, morbidity, clinical prognosis, and mortality. The results of the current scoping review showed a trend of high viral load in the first week of symptom onset with an approximate count of 5.2 log_10_ copies/mL and a subsequent decline in the levels thereafter.^30^ Concurrently, Wolfel et al. reported high pharyngeal virus shedding with average value of 6.76 × 10^5^ copies/whole swab (>5.5 log_10_) in NPS and throat swab samples of COVID-19-positive patients during the first week of symptoms, with a peak on fourth day and a fall in viral load after day 5.^33^ This pattern of rise and subsequent decline of viral burden in throat swabs was also found in a single study included in the current review, which showed full concordance in paired salivary and throat swab samples.^26^

Ct value in the RT-PCR test has for long been considered to be relatively accurate for viral load determination. One of the studies of the current review showed a mean Ct value of 27.16 +/− 3.07, in the initial one week of symptom onset.^17^ This was concordant with aggregated Ct values in NPS by Zou et al., where lower Ct values/higher viral load were found in the initial 1 week of symptom onset.^34^ The Ct value was also found in positive correlation with days from symptom onset in a single cross-sectional study collecting saliva and NPS samples from the COVID-19 screening clinic.^28^ A similar decrease was noted in the viral load of sputum and swab (NPS and throat swab) samples.^33,34^

Detection of active virus in salivary/respiratory sample have been reported in studies for average 18–20 days in mild and severe disease patients.^23,30^ Wolfel et al.^33^ reported the isolation of the live virus in the first week of symptoms in a high percentage of samples [swabs (16.66%), sputum (83.33%)] and not isolated after day 8, although viral load was high. This study showed longevity of upper respiratory swab samples until 28 days,^33^ similar to a single case report of a 71-year-old male patient where the virus was detected until 37 days in saliva.^18^ The higher viral load and duration of the SARS CoV-2 virus in older age is suggested as being related to immunosenescence and a greater level of ACE2, a potent novel CoV receptor.^35,36^ The salivary viral load can be further influenced by the level of ACE2 receptors in various sites of the oral mucosa, epithelial cells of salivary glands, and lung alveoli.^17,23,24^

On correlating disease severity with viral load, studies on salivary samples have indicated higher viral load in severe than in mild disease.^23,30^ A study on upper respiratory tract samples also supported these findings with mean Ct values lower in severe disease patients than mild–moderate disease by 2.8 (95% confidence interval (CI), −2.4 to 8.0) and 2.5 (95% CI, −0.8 to 5.7) in nasal and throat swabs, respectively.^34^

Sex predilection proved greater disease severity and prolonged duration of the virus in males compared to females,^23^ as was reported in infection with other pathogenic CoVs (SARS and MERS).^37^ This difference was attributed to lower immune status in men than in women owing to the difference in hormone levels.^38^

Gargling with chlorhexidine compounds (0.12%, 15 mL) for suppression of lipid-enveloped CoVs has been proposed in one of the studies, where a transient decrease in salivary viral load was reported for 2 h, which regained 2–4 h post-mouthwash.^19^ Although this was a single study on two hospitalised patients, the effect of mouthwashes on SARS-CoV-2 deserves a separate study and review as work has been progressing on mouthwash use in COVID-19, both in hospital and community set-up.

Possibility of self-collection of the saliva specimen is a major advantage over NPS, thus reducing the risk of contamination to HCWs and being non-invasive and comfortable for the patient.^16,18,19,24,25,27–30,32^ Time and cost reduction in saliva compared to NPS sampling has been reported as 1.38- and 2.09-fold, respectively, with mean saliva sample collection time of 114 s and mean cost for a single sample of $1.16.^39^ A comparison of NPS and saliva has been made in multiple studies in the current scoping review, where saliva has proved equally effective as a diagnostic specimen.^16,17,19,32^ Few studies showed positive salivary samples even after NPS was negative,^16,17,21,24^ and a single study reported higher levels of viral titres in saliva than in NPS.^16^ One study has proposed that two negative NPS should be supplemented with an additional negative salivary sample at the time of hospital discharge.^17^

A very recent study by Wyllie et al.^16^ found a higher percentage of viral positivity in saliva compared to NPS in 1–5 days and 6–10 days from COVID-19 diagnosis in 70 hospitalized pateints, whereas samples collected after 11 days found the percentages reversed. The viral titres were also significantly higher in saliva than in NPS in the first available samples.^16^

POCT, used in two studies in the current review, is known to assist in rapid clinical decisions and decrease variations in specimen handling. POCT by the Xpert assay in one study showed 84.5% (49/58) positive paired samples of saliva and NPS^32^ and another study a positive percent agreement of 96% (86.02–99.5%, 95% CI) of saliva and NPS^25^ with no statistically significant difference. Although the viral load in saliva is less compared to NPS,^19,20,24,25,27,28,32^ a few paired sample studies showed detection in saliva and not in NPS; therefore, saliva is suggested to be used as a viable first-line screening test in low resources, multiple environments, and ambulatory at-risk patients.^28^

Salivary antibody surveillance as a non-invasive alternate for serological testing has shown some evidence in the current scoping review. Salivary antibodies are shown to rise ≥10 days after symptom onset, with a high sensitivity and specificity of salivary IgG (anti-RBD).^29,30^ Consistency in temporal kinetics of Igs in serum and saliva (IgM, IgG, IgA) was also observed.^29^ Hence, salivary antibody profiling can be proposed for widespread community-based monitoring of SARS-CoV-2.

Meanwhile, newer second-generation RT-PCR kits are being used to minimise the false-negative results in saliva and increase the SARS-CoV2 detection sensitivity, in the range of 67.1–97% and specificity of 100%.^40–42^ Although these studies were beyond the scope of this review, these kits can offer enhanced diagnostic sensitivity in salivary samples, and future studies should focus on these kits.

The conditions for saliva collection—time, temperature, humidity—have not been given due importance in the studies. One study mentioned higher viral load and higher sensitivity and specificity of SARS-CoV-2 virus in EMSS compared to DSS,^18^ which can be attributed to decreased viral clearance due to cessation of salivary outflow during sleep in early morning samples.^43^ Additionally, DSS can show increased salivary volume and decrease in inhibitory agents to RT-PCR like food, haemoglobin, and various body fluids.^44^

One study reported the rapid spread of infection in local family clusters with the percentage of confirmed cases increasing from 7.7% between days 22 and 32 to 93.1% between days 33 and 42. In addition, authors suggested the importance of prevention of nosocomial infection mentioning strict criteria of isolation to prevent transmission in airborne infection isolation rooms or a minimum 1 m distance between patients for contact, droplet, and airborne precautions. Practices like the discussion of concerns and education of staff in open forums or update of infection control measures were also mentioned.^22^

This scoping review also brings forth some new interesting findings about oral symptoms, which may be of particular interest to dentists and also could prove to be initial warning signs of COVID-19. These oral symptoms like dry mouth, amblygeustia, dryness, and inflammation of the mouth and submandibular lymph nodes enlargement can be attributed to high ACE2 in tongue epithelial cells, providing a possible entry route to SARS-CoV-2, besides being present in salivary glands.^31^ Thus oral symptoms and hygiene can attain a crucial role in diagnosing and preventing COVID-19 and requires further exploration.

### Limitations of the study

Although the present review highlights the available evidence in the viability of saliva as a clinical specimen for diagnosis of SARS-Cov-2, the studies are not without limitations. This review included case reports in the review^18–21^ as they provide excellent insights into some new domains of COVID-19 diagnostics and treatment aspects, like the use of chlorhexidine mouthwash protocol, EMSS better than daytime saliva, detection of virus till 37 days in an old male patient with allergic rhinitis, and viral titres in neonate higher than in mother and persisting in stool longer than respiratory samples.

Keeping in view the current pandemic crisis, the initial diagnostic criteria and patient classification were understandably heterogeneous and non-standardised. There is not enough evidence whether saliva in asymptomatic infected people would carry viral RNA to enable molecular detection as only one study had a control group of asymptomatic HCWs. The majority of the studies were single-centre COVID-19 cohorts, which presented a risk of bias and could overinflate estimates of viral load. Serial salivary samples for temporal evaluation were present in a few studies, but there was a lack of uniformity in the day of salivary sample collection. Hence, the influence of days of symptom onset on salivary viral load remains unresolved. This scoping review also highlights gaps in protocols for saliva sample collection.

## Conclusions

Critical appraisal of literature has brought forth the following inferences:

1. Studies evaluating viral load in paired NPS/saliva samples or serial saliva samples have documented high SARS-CoV-2 viral load in saliva (>5 log10 average) in the first week of symptom onset.
2. Studies evaluating SARS-CoV-2 in saliva with grades of disease severity have shown a significantly increased median duration of virus (2–3 weeks) and higher viral load in patients of severe disease, with or without comparison to mild disease.
3. Studies evaluating N2 and E gene targets in saliva depicted higher detection rate and lower viral load (greater Ct value) in the former and, between ORF1ab and nCoV-N gene, greater detection of N gene.
4. A positive correlation of peak viral log10 value was found with age, more so above 60 years. Sex predilection for the duration of the virus in saliva favoured males more than females, although some studies have negated any correlation with age, sex, or comorbidities.
5. While majority of studies evaluating viral load in paired saliva/NPS samples found a greater sensitivity in NPS than in saliva, few studies reported positive results in saliva, not in NPS, on the same day, which needs further exploration and verification.
6. Studies evaluating the accuracy of POCT in saliva showed high positive percent agreement between PCR-positive NPS and saliva samples.

The findings of this scoping review suggest that saliva can be implored as a viable adjunct specimen in the initial screening of SARS CoV-2 in community or hospital set-up.

### Recommendations

We recommend that future studies on saliva should consider a robust research protocol keeping in view the limitations of earlier studies.

1. Saliva collection protocol for quality assurance should include a serial sampling of saliva, and preferably early morning saliva, before brushing and eating. The saliva should be collected with passive drool, void of coughing. The saliva collection can be either clinician supervised self-collection or with recorded (video/audio) instructions and remote monitoring.
2. Studies should consider a control asymptomatic or healthy group matched for age and sex.

Further studies should as well address the gaps in existing knowledge, notably viral load in the saliva of asymptomatic patients, viral load in correlation with comorbidities and treatments, oral symptoms, age, and sex considerations. Investigation on the presence of salivary COVID-19 viral load in children is also warranted.
